# Microbial identification from traumatized immature permanent teeth with periapical lesions using matrix-assisted laser desorption/ionization time-of-flight mass spectrometry

**DOI:** 10.1186/s12903-022-02562-y

**Published:** 2022-12-31

**Authors:** Pervine H. Sharaf, Rania M. El Backly, Raef A. Sherif, Ashraf M. Zaazou, Soad F. Hafez

**Affiliations:** 1 Conservative Dentistry Department, Faculty of Dentistry, Alexandria University, Alexandria, Egypt, Endodontic Specialist, Ministry of Health, Alexandria, Egypt; 2 Endodontics, Conservative Dentistry Department, Faculty of Dentistry, Alexandria University, Alexandria, Egypt; 3 Faculty of Dentistry, King Abdel Aziz University, Jeddah, Saudi Arabia; 4 Medical Microbiology and Immunology Department, Faculty of Medicine, Alexandria University, Alexandria, Egypt

**Keywords:** MALDI-TOF MS, Microbial identification, Periapical lesions, Regenerative endodontics, Traumatized immature permanent teeth

## Abstract

**Background:**

This study aims at identifying the microbiota in traumatized immature permanent teeth with periapical lesions using Matrix-assisted laser desorption/ionization time-of-flight mass spectrometry (MALDI-TOF MS).

**Methods:**

The study included 16 immature maxillary central incisors with periapical lesions in 13 patients. Field decontamination and negative control samples were performed before and after access cavity preparation. Root canal samples were taken using sterile stainless-steel hand files following field decontamination. In-office inoculation and pure sub-cultures were performed. Bacterial isolates were prepared for MALDI-TOF MS (Bruker, Billerica, MA USA) analysis using the formic acid extraction method. A comparison of the prevalence of isolated microorganisms was done using a one-sample chi-square test. Comparisons between identified microbial species with the, cone beam computed tomography periapical index (CBCT PAI) scores and lesion volume were also conducted. The Chi-square test was applied to investigate the association between the categorical variables .

**Results:**

Out of the forty isolates recovered from the 16 traumatized teeth included in the present study with the mean patients’ age of 10.93 ± 1.77, 37 isolates were reliably identified by MALDI-TOF MS. Twelve teeth (62.5%) were polymicrobial. The recovered bacteria belonged to five phyla, 15 genera and 25 species. Firmicutes were the predominant phylum (*P* < 0.001) over Bacteroidetes, Proteobacteria, Actinobacteria and Fusobacteria. Gram positive bacteria were significantly more prevalent than Gram negative (*p* = 0.03). Facultative anaerobes were the most prevalent (*P* < 0.001) compared to the obligate anaerobes and the obligate aerobes. The latter were the least prevalent. Statistically, significant differences existed in the comparison between CBCT PAI scores according to bacterial gram staining.

**Conclusion:**

Traumatized immature permanent teeth with periapical lesions showed a significant predominance of Gram-positive facultative anaerobes. MALDI-TOF MS provided accurate identification of numerous viable endodontic microbes.

## Background

Persistent infection has been shown to be the cause of failed regenerative endodontic treatment (RET) in 79% of unsuccessful cases [1]. This treatment modality has recently risen as the first treatment choice for immature necrotic permanent teeth. Bacterial products have been found to compromise cell viability, proliferation, and mineralization capacity of stem cells from the apical papilla (SCAPs) [2]. These products also decreased the release of growth factors essential for regeneration [3]. Additionally, a residual infection may interfere with true dentin-like tissue regeneration supporting a more osteogenic rather than dentinogenic differentiation of mesenchymal stem cells [4]. This adds emphasis to the need to design effective antimicrobial strategies to target the unique microbial communities present in immature infected teeth [5, 6].

Regarding the level of bacteria following RET in immature traumatized teeth, de-Jesus-Soares et al. 2020 [7] correlated microbiological data retrieved from cases treated with RET with clinical and radiographic findings after 12–48 months. While they found a significant reduction of bacterial levels after irrigation and then following intracanal medication with either triple antibiotic paste (TAP) or calcium hydroxide with chlorhexidine (CHP) there was no significant difference between these groups regarding periapical healing. Additionally, despite a low level of residual bacteria, a negative impact on the thickness of dentinal walls was observed.

Although most cases indicated for RET present with a history of trauma, there is a scarcity of knowledge on the microbiota of traumatized immature necrotic teeth. Studies by Baumotte et al. 2011 [5] and Nagata et al. 2014 [6] used culture and molecular methods, respectively. Both methodologies identified selected or pre-targeted microbiota of traumatized immature necrotic permanent teeth. Despite their statements about the similarity of microbiota in immature and mature teeth, they highlighted some differences. This calls for more studies involving immature teeth. Baumotte et al. 2011 [5] using selective media for *Enterococcus* and *yeast* species conducted a culture quantitative analysis. The specimens showed black pigmented species in (21.05%) and *Enterococcus* species in (5.25%). Whereas, Nagata et al. 2014 [6] used polymerase chain reaction assay (16SrRNA) to identify cultivable bacteria, examining *Actinomyces naeslundii* in (66.67%) of the root canals, while (33.34%) showed *Porphyromonasendodontalis*, *Parvimonas micra*, and *Fusobacterium nucleatum*.

Despite the added precision of molecular methods, many of them still have clear limitations. These include, but are not limited to, the possibility of producing misleading results due to the traces of DNA contamination or non-specific annealing. This is beside the general inability of these techniques to distinguish between viable and non-viable microorganisms except for PCR combined with the use of cell viability dyes or reverse-transcriptase PCR to detect pre-messenger RNA. They also aim at a limited number of pre-targeted pathogens using specific primers or probes [7–9]. The recent advent of metagenomic next-generation sequencing (mNGS) has highly improved the scalability and the ability of molecular techniques to identify all potential pathogens, yet it is expensive and its clinical utility is still to be established [10].

Matrix-assisted laser desorption/ionization time-of-flight mass spectrometry (MALDI-TOF MS) has revived the field of culture-based clinical microbiology by allowing clinical laboratories to identify microorganisms as soon as visible growth is observed. It is a soft ionization approach that depends on bacterial identification via peptide mass fingerprinting (PMF) which comprises comparing the mass spectra (MS) of the unknown organism which is generated through desorption of bacterial proteins in the form of ions with the MS of known microbial isolates contained in the database. The process is rapid, sensitive, and economic. It characterizes a wide variety of microorganisms including bacteria, fungi and viruses. It has been shown that MALDI-TOF MS identification of anaerobic bacteria is more reliable than conventional biochemical methods by 24% at the species level [11]. MALDI-TOF MS is often also able to distinguish between closely related bacterial species phenotypically, biochemically, or genetically because it measures the microbial proteins overcoming the difficulties encountered by traditional or molecular methods in such identifications [12]. Furthermore, culture-dependent MALDI-TOF MS identifies viable bacteria [13].

Therefore, the aim of the present clinical study was to identify the microbiota in traumatized immature permanent teeth with periapical lesions using (MALDI-TOF MS).

## Methods

### Study design

This prospective observational study included 16 maxillary central incisors with radiographic evidence of immature roots and periapical lesions in 13 patients. Cases were recruited from the clinics of the Conservative Dentistry and Pediatric Dentistry Departments, Faculty of Dentistry, Alexandria University, Egypt.

All procedures performed were in accordance with the ethical standards of the Research Ethics Committee, Faculty of Dentistry, Alexandria University and with the 1964 Helsinki Declaration and its later amendments or comparable ethical standards. The clinical work was performed after the approval of the Research Ethics Committee, Faculty of Dentistry, Alexandria University (IRB 00,010,556)-(IORG 0,008,839) on 4/7/2018. Written informed consent was signed by the guardian of the patient.

The inclusion criteria included both male and female patients ranging in age from eight to eighteen years presenting with traumatized immature permanent upper anterior teeth showing periapical lesions on periapical radiographs indicated for RET. The time between the trauma and the first visit varied from 6 months to two years.

Cone-beam computed tomographic assessment was performed for all patients to correlate between accurate lesion size according to Estrela et al.’s Cone-beam computed tomographic periapical index score (CBCT PAI) [14] and identified microbial species.

Exclusion criteria included teeth with root fracture or periodontal disease or orthodontic wires or brackets. Patients with a history of allergy to any medication or bleeding disorders or any medical illness or taking antibiotics during the previous 3 months were excluded.

Cone-beam computed tomographic images were taken using Veraview epocs 3D R 100 (J Morita Corp, Koyoto, Japan) operating at 90 kV and 8 mA with an exposure time of 9.4 s, a field of view of 40 × 40, and voxel size of 0.125 mm. Lesion size was determined according to Estrela et al. Cone-beam computed tomographic periapical index score (CBCT PAI) [14] and lesion volume was calculated with the medical image computing platform OsiriX MD software. Furthermore, comparisons between identified microbial species according to CBCT PAI as well as lesion volume were conducted.

### Tooth samples

Before sampling, removal of plaque and calculus was performed. Mepivacaine hydrochloride 3% local anaesthesia was given as infiltration opposite to each treated tooth (Septodont, Cedex, France). The tooth was then isolated with a rubber dam (Sanctuary, Selangor, Malaysia). The dam, the clamp, and the tooth were disinfected with 2.5% Sodium hypochlorite (NaOCl) and Povidone iodine 10% (El Nile co, Cairo, Egypt) for 30 s each. To ensure the sterility of the operation field, negative control samples were swabbed from the crown and dam and then plated on blood agar. Field inactivation was not required since samples were immediately placed in thioglycollate broth (Oxoid, Cheshire, England) in 2 ml screw-capped vials, which is known to be a reducing agent and an enrichment broth that supports the growth of anaerobes, aerobes, microaerophilic, and fastidious microorganisms, requiring no special equipment in the dental clinic [15–17]. Then, the access cavity was prepared with a sterile high-speed diamond bur without the use of water spray but under manual irrigation with sterile saline solution. Disinfection procedures and sterility control samples were repeated before sampling.

Minimal instrumentation of the canal walls to the estimated working length was performed using a sterile stainless-steel hand file of 2% taper matching the size of the canal (Dentsply Maillefer, Ballaigues, Switzerland). This instrument together with the dentine debris represented the sample obtained [18] ensuring the collection of biofilms possibly penetrating deep in the dentine of immature permanent teeth [19]and reaching the apical third whereas impregnated paper points cannot always reach these depths [20]. Direct in-office (chairside) inoculation was performed to avoid the death of sensitive anaerobes [21]. Then, the full length of the cutting blade of the file was moistened with freshly prepared fluid thioglycolate broth in 2 ml screw-capped vials.

The file carrying the dentine swarf was inoculated sequentially onto anaerobic and aerobic freshly prepared blood agar plates (Blood Agar base + 5% sterile blood) (Oxoid, Cheshire, England) creating the first streak. Next, a sterile loop was used to streak five lines from the primary inoculum. Then, the streak pattern was repeated three more times; each streak was taken from its previous streak, each using a new sterile loop, giving rise to five growth areas in total (the primary inoculum plus the four streaked areas) to guarantee recovery of separate colonies. The streak plate technique is the most popular method for isolating specific colonies from a sample containing a mixture of microorganisms. Sterile loops were used for each streak area to obtain uncontaminated microbial cultures and to ensure the picking up of isolated colonies [22, 23].

The anaerobic plates were placed in an anaerobic jar ensuring an anaerobic atmosphere through commercially available anaerogenerating gas packs (Oxoid, Cheshire, England), while, aerobic plates were incubated in an ambient atmosphere. Plates were incubated at 37º C for 48 h as it has been demonstrated that for the best chances of reliable identification, colonies should not be older than 2 days, with even the most sensitive species identified with a score > 2.0 after this time [13]. Reliable species identification was obtained after 48 h of incubation for gram-negative anaerobes and after 72 h for gram-positive anaerobes [24].

After the predetermined incubation period for aerobic and anaerobic cultures, pure bacterial isolates were prepared for MALDI-TOF MS analysis using the formic acid extraction method according to Schulthess’ et al. protocol [25].

### MALDI-TOF MS Analysis

Briefly, the protocol includes suspension of a 1-μL loopful of bacterial growth in 300 μL high-performance liquid chromatography-grade water (Sigma-Aldrich, St. Louis, MO, USA) and 900 μL pure ethanol (Sigma-Aldrich, St. Louis, MO, USA). After centrifuging the tube at 13,000g for 2 min in a centrifuge machine (Thermo Electron Waltham, MA, USA), the supernatant was removed and the pellet was left to dry. Then, it was suspended in an equal amount of 70% formic acid (Amresco, Solon, OH, USA) and 100% acetonitrile (Sigma-Aldrich, St. Louis, MO, USA). The mixture was then centrifuged at 13,000g for 2 min. One microliter of the supernatant was added to each spot on a matrix-assisted laser desorption/ionization target plate in duplicate and was left to dry. After drying of the extract, 1 μL matrix solution was prepared from 25 μL pure trifluoroacetic acid (Alfa Aesar, Haverhill, MA, USA), 475 μL high-performance liquid chromatography-grade water, 500 μL acetonitrile, and 10 mg a-cyano-4-hydroxycinnamic acid (Sigma-Aldrich, St. Louis, MO, USA), was added to each spot. After air-drying, the matrix-assisted laser desorption/ionization target plate was inserted into a Microflex LT mass-spectrometer (Bruker, Billerica, MA USA) using the manufacturer's settings and the mass spectra generated were analyzed using FlexControl software (version 3.0) (Bruker, Billerica, MA, USA). The identification score cut-offs accepted for reliable identification to the species level, and probable genus level recommended by the manufacturer of MALDI-TOF MS were: 2.0–3.0, and 1.700–1.999, respectively, while scores < 1.700 were considered as no identification. In the present study, the proposed scores of accuracies, by Scott JS et al. 2016 [26] ≥ 1.5 for genus level identification and ≥ 1.7 for species level were adopted.

The current study followed the guidelines for STrengthening the Reporting of OBservational studies in Epidemiology (STROBE).

### Statistical analysis

Data were analyzed using IBM SPSS for Windows (Version 23.0) and significance was set at *p*-value < 0.05. Frequencies and percentages were calculated for all qualitative variables. A comparison of the prevalence of isolated microorganisms was done using a one-sample chi-square test. The Chi-square test was applied to investigate the association between the categorical variables. For continuous data, they were tested for normality by the Shapiro–Wilk test. Quantitative data were expressed as mean and standard deviation. For not normally distributed quantitative variables Mann Whitney test was used to compare two groups while Kruskal Wallis test was used to compare between more than two groups. The bacterial network was visualized using Gephi software (Version 0.9.2) where nodes represent bacterial genera and edges reflect the repeated isolation of these genera together.

## Results

The samples were collected from 16 teeth out of thirteen patients (12 teeth in nine males and four teeth in four females) with a mean age of 10.93 ± 1.77. All the teeth were traumatized immature permanent maxillary central incisors with periapical lesions. All the teeth were at least a stage nine according to Nolla's stages of tooth development with completed root and open apex [27]. Periapical diagnosis was asymptomatic apical periodontitis and chronic apical abscess in 11 and five teeth, respectively. While, the pulpal diagnosis was necrosis in 12 teeth(75%), previous treatment in three teeth (18.75%), and previous initiated treatment in one tooth (6.25%). (Table 1).

**Table 1:** Patient demographics and characteristics of affected teeth included in the present study

Pt	Age	Gender	Diagnosis	Identification	Number of bacterial species	CBCT PAI	E/D	Volume (mm^3^)
1	**10**	**M**	**Pulp necrosis &asymptomatic apical periodontitis**	**Slackia exigua** **Fusobacterium nucleatum** **Prevotella oralis** **Selenomonas infelix**	**4**	**5**		**1.419**
2	**12**	**M**	**Previously treated &asymptomatic apical periodontitis**	**Streptococcus sanguinis**	**1**	**4**		**2.8348**
3	**12**	**M**	**Pulp necrosis & chronic apical abscess**	**Lactobacillus paracasei**	**1**	**5**		**3.430**
4	**11**	**F**	**Pulp necrosis &asymptomatic apical periodontitis**	**Staphylococcus aureus** **Enterococcus faecalis**	**2**	**4**	**D**	**1.486**
5	**13**	**M**	**Pulp necrosis &asymptomatic apical periodontitis**	**Streptococcus sanguinis** **Enterococcus faecalis** **Staphylococcus epidermidis**	**3**	**5**	**D**	**3.345**
6	**8**	**M**	**Pulp necrosis & chronic apical abscess**	**Staphylococcus warneri**	**1**	**5**	**E**	**3.549**
7	**12**	**M**	**Pulp necrosis &asymptomatic apical periodontitis**	**Enterococcus faecalis** **Bacteroides ovatus** **Prevotella negrescens**	**3**	**5**		**2.262**
8	**11**	**F**	**Previously initiated &asymptomatic apical periodontitis**	**Clostridium sphenoides**	**1**	**5**	**D**	**4.439**
9	**10**	**M**	**Pulp necrosis &asymptomatic apical periodontitis**	**Streptococcus constellatus**	**1**	**4**		**1.986**
10	**10**	**M**	**Pulp necrosis &asymptomatic apical periodontitis**	**Cupriavidus metallidurans** **Prevotella denticola** **Prevotella negrescens** **Streptococcus anginosus**	**4**	**5**	**D**	**3.156**
11	**10**	**F**	**Pulp necrosis &asymptomatic apical periodontitis**	**Streptococcus pneumoniae** **Streptococcus mitis**	**2**	**4**	**D**	**1.064**
12	**12**	**M**	**Previously treated &asymptomatic apical periodontitis**	**Bacillus cereus**	**1**	**4**		**9.2769**
13	**9**	**F**	**Pulp necrosis & chronic apical abscess**	**Enterococcus faecalis** **Streptococcus anginosus** **Prevotella denticola** **Prevotella negrescens**	**4**	**5**	**D**	**3.414**
14	**14**	**M**	**Pulp necrosis &asymptomatic apical periodontitis**	**Enterococcus faecalis** **Streptococcus sanguinis** **Mycobacterium genavense** **Bacillus atrophaeus** **Listeria grayi**	**5**	**4**		**2.764**
15	**9**	**M**	**Pulp necrosis & chronic apical abscess**	**Acinetobacter calcoaceticus** **Staphylococcus epidermidis** **Prevotella buccae**	**3**	**5**	**D**	**3.903**
16	**13**	**M**	**Previously treated & chronic apical abscess**	**Salmonella typhi** **Prevotella denticola** **Bacillus licheniformis** **Klebsiella pneumoniae**	**4**	**5**		**1.140**

CBCT PAI:Cone-beam computed tomographic periapical index

E/D: Expansion/Destruction

(mm^3^): cubic millimeter

The number of microorganisms identified per canal ranged from one to five. The average (mean) number of species identified per canal was 2.5 ± 1.414.

From the 16 teeth included in the present study,40 isolates were recovered. Only 37 isolates (species)showed reliable identifications by 92.5% according to the score cut-off recommended by the manufacturer of MALDI-TOF MS modified by the proposed scores of Scott JS et al. 2016 [27]. (Table 2). The 37 isolates belonged to 15 genera and 25 different species.

**Table 2:** Isolated bacteria and their MALDI-TOF MS identification score

**Pt**	**ID score of recovered bacteria**			
	**score ≥ 2**	**Score ≥ 1.7–1.9**	**score < 1.7–1.5**	**Score < 1.5**
**1**	Slackia exigua Fusobacterium nucleatum Selenomonas infelix	Prevotella oralis		
**2**	Strept sanguinis			
**3**		Lactobacillus paracasei		
**4**	Enterococcus faecalis	Staph aureus		
**5**	Enterococcus faecalis Staph epidermidis	Streptococcus sanguinis		
**6**	Staph warneri			
**7**	Enterococcus faecalis Bacteroides ovatus	Prevotella negrescens		
**8**			Clostsphenoides (Clostridium sp.)	
**9**	Strept constellatus			
**10**	Cupriavidus metallidurans Prevotella denticola Prevotella negrescens Strept anginosus			
**11**	Strept pneumoniae Streptococcus mitis			
**12**			Bacillus cereus (Bacillus sp.)	
**13**	Enterococcus faecalis Strept anginosus Prevotella denticola Prevotella negrescens			
**14**	Enterococcus faecalis Strept sanguinis			Mycob. genavense Bacillus atrophaeus Listeria grayi
**15**		Prevotella buccae	Acinetobacter calcoaceticus (Acinetobacter sp.) Staph epidermidis (Staphylococcus sp.)	
**16**	Salmonella typhi Prevotella denticola Bacillus licheniformis Klebsiella pneumoniae			
	**27 (67.5%)**	**6 (15%)**	**4(10%)**	**3 (7.5%)**

**Note:** *Eight isolates (20%) belonging to the *Streptococcus* genera were isolated from 7 teeth (43.75%)

In one tooth, *Streptococcus pneumoniae*&*Streptococcus mitis* were concomitantly isolated

^**^ Eight isolates (20% of total isolates) belonging to the *Prevotella* were detected in six teeth (37.5%) as *P denticola*&*P negrescens* were concomitantly detected in two teeth

^***^ Five *Enterococcus faecalis* five isolates were isolated (12.5% of total isolates), from five teeth (31.25%)

^****^The identification of four isolates with scores < 1.7—1.5 was accepted to the genus level only

^*****^The three isolates (7.5%) with scores < 1.5 were considered unidentified

**Fig. 1:**
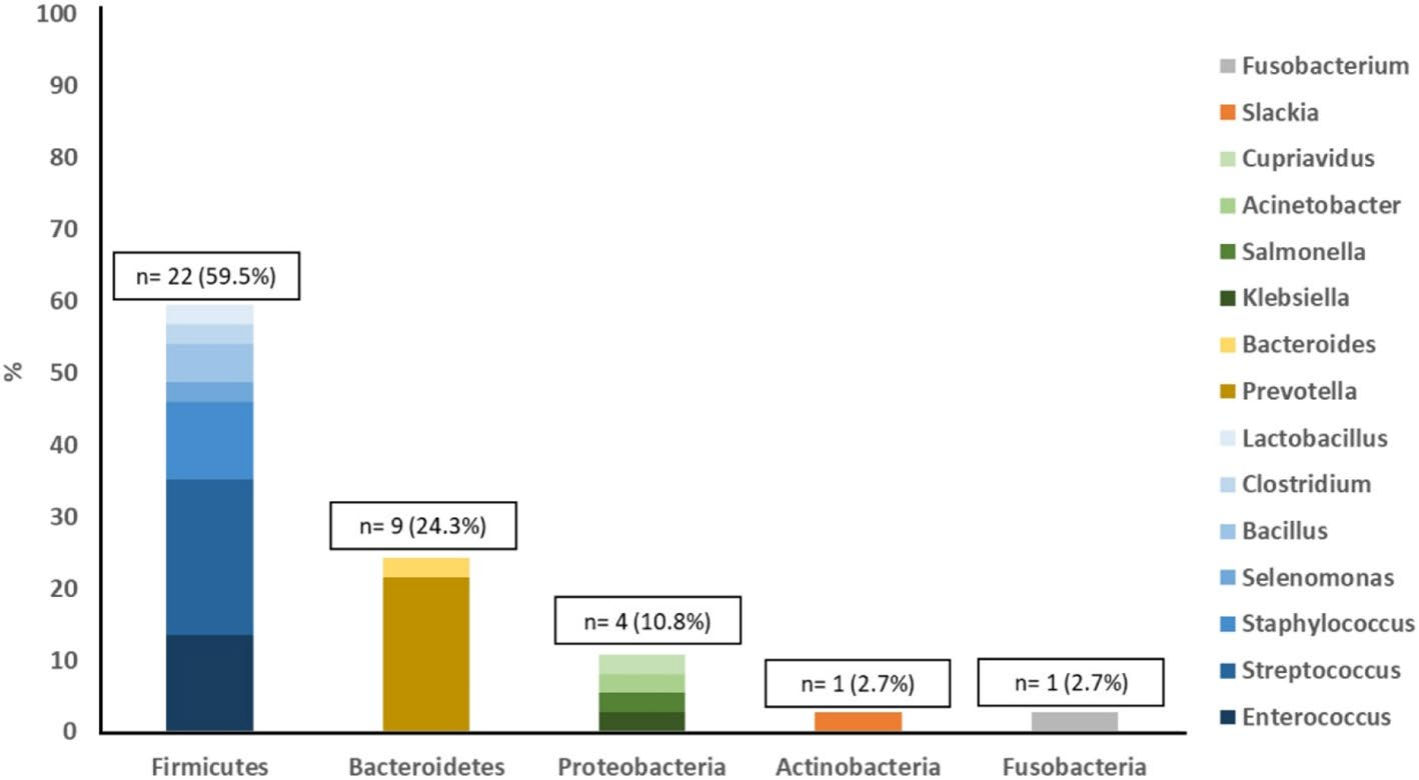
Bacteria isolated from the root canals in the present study distributed according to their phyla

Five bacterial phyla were identified in the 37 species with acceptable identification score: Firmicutes 59.5% (22 species), Bacteroidetes 24.3% (9 species), Proteobacteria 10.8% (4 species), Actinobacteria 3.7% (one species), and Fusobacteria 3.7% (one species). The prevalence of Firmicutes was statistically significant (*P* < 0.001).Fig. 1 shows the different genera included in each phylum in this study.

Regarding the gaseous requirements of the 37 isolates only obligate anaerobic bacteria were isolated in two teeth (12.5%). While only facultative anaerobes were isolated in nine teeth (56.25%). In five teeth (31.25%) both obligate and facultative anaerobes were isolated; in one of which the only aerobic genera recovered was concomitantly isolated. Comparing the number of isolates, a significant difference existed in the prevalence of obligate anaerobes, aerobes and facultative anaerobes (*P* < 0.001), with the highest prevalence for facultative anaerobes with 23 isolates (62.16%), followed by obligate anaerobes with 13 isolates (35.14%) and one obligate aerobe isolate (2.7%). (Table 3).

**Table 3:** Bacterial genera isolated in the present study, their Gram-staining characteristics and gaseous requirements

**Bacteria morphology**	**Obligate anaerobes**	**#**	** Obligate** **aerobe**	**#**	**Facultative anaerobes**	**#**	**Total**	**P value**
**Gram** **positive cocci**					Streptococcus	8		
					Staphylococcus	4		
					Enterococcus	5		
**Gram** **negative cocci**	=============				====================			
**Gram negative coccobacilli**			**Acinetobacter spp.**	1				
**Gram** **positive rods**					Lactobacillus	1		
	Slackia	1			Bacillus	2		
	Clostridium	1						
**Gram -negative rods**	Fusobacterium	1			Salmonella	1		
	Prevotella	8			Klebsiella	1		
	Selemonas	1			Capriavidus	1		
	Bacteroides	1						
**Number (%) from** **Total Genera**	6 (40%)	==	1 (6.7%)	1	8 (53.3%)	==	15 100%	
**Number (%) of teeth positive (%)**	7 (43.75%)		1 (6.25%)		14 (87.5%)		16	**<**0.001***
**Total isolates Number** **(%)**	13 (35.14%)		1 (2.7%)		23 (62.162%)		37 (100%)	**<**0.001***

^*^Some species can also be strict aerobes

*Mycobacterium genavense, Bacillus atrophaeus*, and *Listeria gravi* are not included due to their low MALDI-TOF MS identification score (< 1.5)

^**^ in two teeth (12.5%) only obligate anaerobic bacteria were isolated

^***^ In 9 teeth (56.25%) only facultative anaerobes were isolated

^****^ In 5 teeth (31.25%) both obligate and facultative anaerobes were isolated in one of which the only aerobic genera recovered was concomitantly isolated

^*^statistically significant at *p*-value < 0.05

**Fig. 2:**
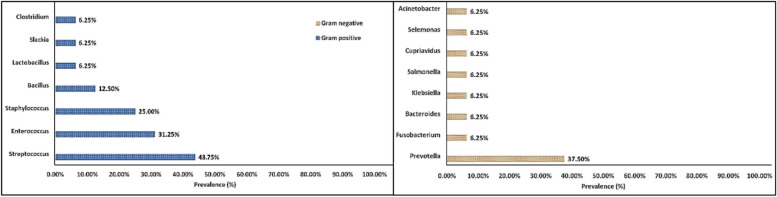
Prevalence of Gram-positive and Gram-negative genera recovered from 16 teeth included in this study

All teeth showed Gram-positive bacteria (100%), whereas Gram-negative bacteria were isolated only from six teeth (37.5%) which displayed mixed Gram-positive & Gram-negative isolates. The prevalence of Gram-positive bacteria was higher than Gram-negative. This difference was statistically significant (*P* = 0.03). Within the Gram-positive genera, the most prevalent genus was *Streptococcus* while within the Gram-negative genera, the most prevalent was the *Prevotella*, with no statistical significance for either. (Fig. 2).

**Fig. 3:**
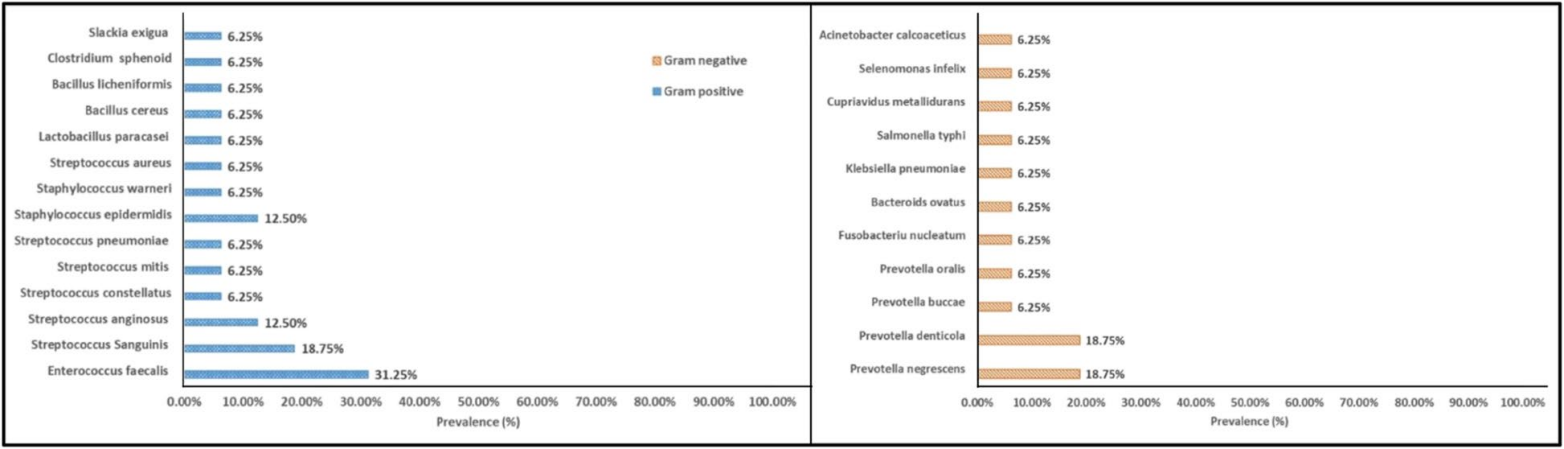
Prevalence of Gram positive and Gram negative species

Regarding the Gram-positive species isolated in the present study, the most prevalent species was *Enterococcus faecalis* while among the Gram-negative species the most prevalent species were *Prevotella denticola* & *Prevotella negrescens* again with no significant differences. (Fig. 3).

Regarding the comparison between microbiota identification according to CBCT results, the CBCT PAI of ten lesions (62.5%) scored five, as the diameter of the periapical radiolucency was greater than 8 mm and six lesions (37.50%) scored four as the diameter of the periapical radiolucency was between 4 and 8 mm. Periapical cortical bone expansion and destruction were seen in one case and seven cases, respectively. The comparison between microbial genera or phyla in accordance with CBCT PAI was not statistically significant (*p*-value > 0.05), nor was the comparison between them according to periapical lesion volume (*p*-value > 0.05). Statistically significant differences existed in the comparison between CBCT PAI scores according to gram stain, with higher scores (score five) associated with gram-negative bacteria. Mixed gram-negative and positive infections scored five while only gram-positive infections scored four and five. (Chi-square testX^2^ = 10.45, Monte Carlo corrected *p*-value P_MC_ = 0.02, statistically significant at *p*-value < 0.05) (Tables 4, 5, 6, 7, 8 and 9).

**Table 4:** Lesion volume according to bacteria genera

		**Mean (SD)** (mm^3^)	**KWT (*p*-value)**
**Lesion volume**	**Acinetobacter**	3.9 (0.00)	H = 14.62 P = 0.40 NS
	**Bacillus**	5.2 (5.7)	
	**Bacteroides**	2.3 (0.00)	
	**Clostridium**	4.4 (0.00)	
	**Cupriavidus**	3.1 (0.00)	
	**Enterococcus**	2.6 (0.8)	
	**Fusobacterium**	1.4 (0.00)	
	**Klebsiella**	1.1 (0.00)	
	**Lactobacillus**	3.4 (0.00)	
	**Prevotella**	2.7 (1.0)	
	**Salmonella**	1.1 (0.00)	
	**Selenomonas**	1.4 (0.00)	
	**Slackia**	1.4 (0.00)	
	**Staphylococcus**	3.1 (1.1)	
	**Streptococcus**	2.4 (1.0)	

*KWT* Kruskal Wallis test, *NS* Non-significant (mm^3^), cubic millimeter

**Table 5:** CBCT PAI according to bacteria genera

	**Score 0**	**Score 1**	**Score 2**	**Score 3**	**Score 4**	**Score 5**	**Score 4D**	**Score 5D**	**Score 5E**	**X**^**2**^** (p value)**
Acinetobacter	0 (0%)	0 (0%)	0 (0%)	0 (0%)	0 (0%)	0 (0%)	0 (0%)	1 (100%)	0 (0%)	X^2^ = 44.08 P_MC_ = 0.83 NS
Bacillus	0 (0%)	0 (0%)	0 (0%)	0 (0%)	1 (50%)	1 (50%)	0 (0%)	0 (0%)	0 (0%)	
Bacteroides	0 (0%)	0 (0%)	0 (0%)	0 (0%)	0 (0%)	1 (100%)	0 (0%)	0 (0%)	0 (0%)	
Clostridium	0 (0%)	0 (0%)	0 (0%)	0 (0%)	0 (0%)	0 (0%)	0 (0%)	1 (100%)	0 (0%)	
Cupriavidus	0 (0%)	0 (0%)	0 (0%)	0 (0%)	0 (0%)	0 (0%)	0 (0%)	1 (100%)	0 (0%)	
Enterococcus	0 (0%)	0 (0%)	0 (0%)	0 (0%)	1 (20%)	1 (20%)	1 (20%)	2 (40%)	0 (0%)	
Fusobacterium	0 (0%)	0 (0%)	0 (0%)	0 (0%)	0 (0%)	1 (100%)	0 (0%)	0 (0%)	0 (0%)	
Klebsiella	0 (0%)	0 (0%)	0 (0%)	0 (0%)	0 (0%)	1 (100%)	0 (0%)	0 (0%)	0 (0%)	
Lactobacillus	0 (0%)	0 (0%)	0 (0%)	0 (0%)	0 (0%)	1 (100%)	0 (0%)	0 (0%)	0 (0%)	
Prevotella	0 (0%)	0 (0%)	0 (0%)	0 (0%)	0 (0%)	3 (37.5%)	0 (0%)	5 (62.5%)	0 (0%)	
Salmonella	0 (0%)	0 (0%)	0 (0%)	0 (0%)	0 (0%)	1 (100%)	0 (0%)	0 (0%)	0 (0%)	
Selenomonas	0 (0%)	0 (0%)	0 (0%)	0 (0%)	0 (0%)	1 (100%)	0 (0%)	0 (0%)	0 (0%)	
Slackia	0 (0%)	0 (0%)	0 (0%)	0 (0%)	0 (0%)	1 (100%)	0 (0%)	0 (0%)	0 (0%)	
Staphylococcus	0 (0%)	0 (0%)	0 (0%)	0 (0%)	0 (0%)	0 (0%)	1 (25%)	2 (50%)	1 (25%)	
Streptococcus	0 (0%)	0 (0%)	0 (0%)	0 (0%)	3 (37.5%)	0 (0%)	2 (25%)	3 (37.5%)	0 (0%)	

X^2^: Chi-square test, P_MC_: Monte Carlo corrected p value, NS: Non-significant

**Table 6:** CBCT PAI according to gaseous requirements of isolated bacteria

	**Score 0**	**Score 1**	**Score 2**	**Score 3**	**Score 4**	**Score 5**	**Score 4D**	**Score 5D**	**Score 5E**	**X**^**2**^ **(*p* value)**
	**N (%)**									
**Obligate anaerobes**	0 (0%)	0 (0%)	0 (0%)	0 (0%)	0 (0%)	7 (53.8%)	0 (0%)	6 (46.2%)	0 (0%)	X^2^ = 10.22 P_MC_ = 0.24 NS
**Facultative anaerobes**	0 (0%)	0 (0%)	0 (0%)	1 (4.3%)	5 (21.7%)	4 (17.4%)	4 (17.4%)	8 (34.8%)	1 (4.3%)	
**Aerobes**	0 (0%)	0 (0%)	0 (0%)	0 (0%)	0 (0%)	0 (0%)	0 (0%)	1 (100%)	0 (0%)	

X^2^: Chi-square test, P_MC_: Monte Carlo corrected p value, NS: Non-significant

**Table 7:** Lesion volume according to gaseous requirements of isolated bacteria

	**Obligate anaerobes**	**Facultative anaerobes**	**Aerobes**	**KWT (*p* value)**
	**Mean (SD)**			
**Lesion volume**	2.5 (1.1)	2.8 (1.7)	3.9 (0.0)	H = 2.00 P = 0.37 NS

*KWT* Kruskal Wallis test, *NS* Non-significant

**Table 8:** Lesion volume according to Gram stain

	**Gram positive**	**Gram negative**	**Mann–Whitney** **(*p* value)**
	**Mean (SD)**		
**Lesion volume**	2.9 (1.7)	2.4 (1.1)	Z = 0.82 P = 0.42 NS

*NS* Non-significant

**Table 9:** CBCT PAI according to Gram stain of isolated bacteria

	**Score 0**	**Score 1**	**Score 2**	**Score 3**	**Score 4**	**Score 5**	**Score 4D**	**Score 5D**	**Score 5E**	**X**^**2**^** (*p* value)**
	**N (%)**									
**Gram Positive**	0 (0%)	0 (0%)	0 (0%)	0 (0%)	5 (22.7%)	4 (18.2%)	4 (18.2%)	8 (36.4%)	1 (4.5%)	X^2^ = 10.45 P_MC_ = 0.02*
**Gram negative**	0 (0%)	0 (0%)	0 (0%)	0 (0%)	0 (0%)	8 (53.3%)	0 (0%)	7 (46.7%)	0 (0%)	

X^2^: Chi-square test, P_MC_: Monte Carlo corrected p value

*statistically significant at *p* value < 0.05

**Fig. 4:**
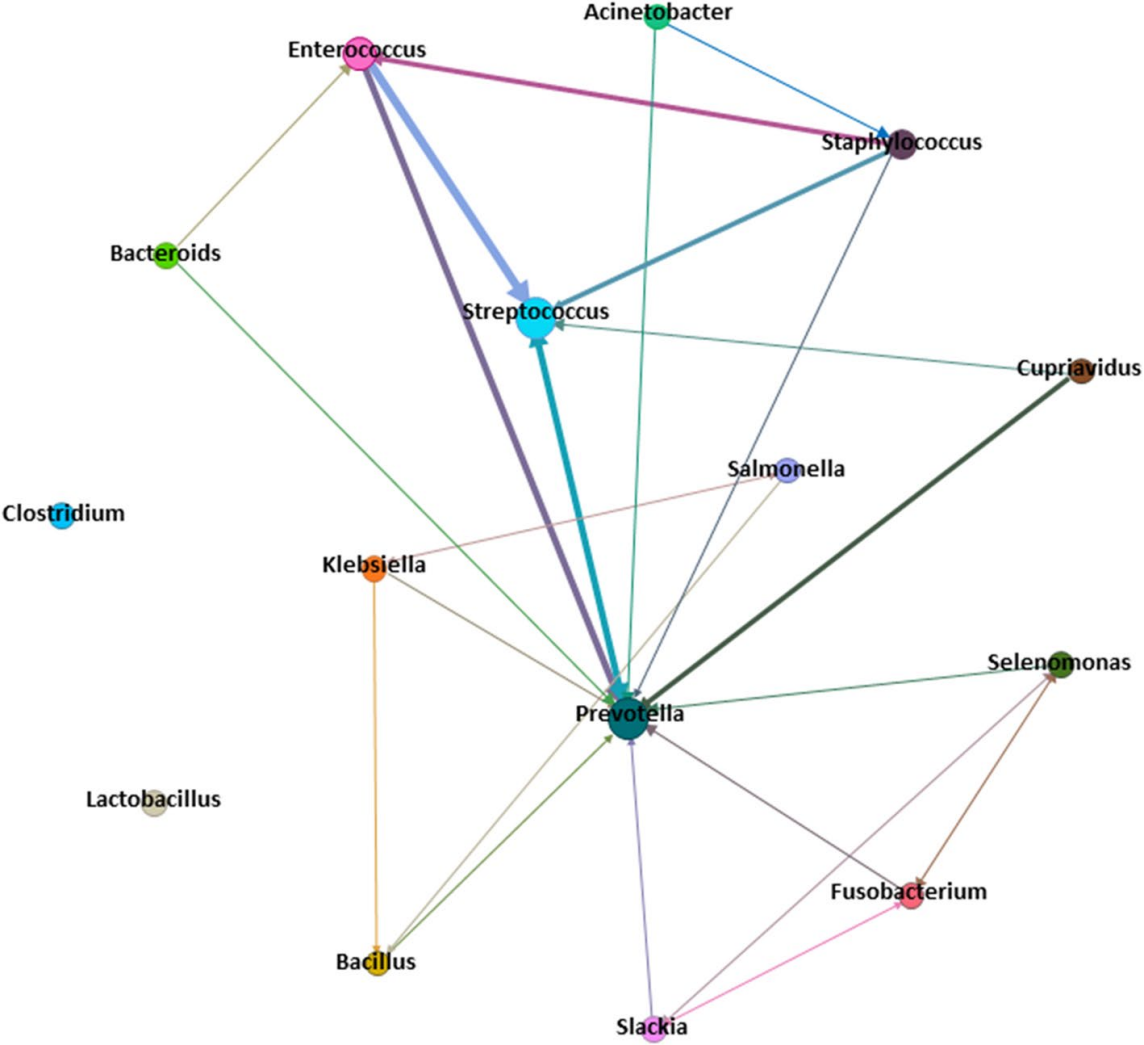
Bacterial network reflecting the co-occurrence patterns of bacteria belonging to the different genera isolated in the present study. (Nodes represent bacterial genera. The thickness of the edges increases according to the repetition of the co-occurrence patterns. The unidirectional arrow points toward the more repeated genera. The bidirectional arrows indicate an equal incidence of genera.)

Upon analyzing the presence of co-occurrence patterns of bacterial genera using bacterial networking, the genus *Prevotella* was encountered with all genera except *Clostridium* and *lactobacillus* which were isolated from monomicrobial lesions. The more repeated genera as demonstrated by unidirectional arrow points were *Streptococcus, Prevotella,* and *Enterococcus*. Indeed, as marked by the size of the nodes, the most abundant genera were *Streptococcus, Prevotella*, and *Enterococcus*. The thick edges between *Streptococcus, Prevotella*, and *Enterococcus* denote the repetition of their co-occurrence. (Fig. 4).

## Discussion

It has been shown that quantity of detected microorganisms in infected root canals plays a role in the healing of periapical lesions; leading to either failure or delayed healing response due to some residual infection [28]. Moreover, the residual infection may impede the thickening of the root following RET [7]. Previous studies showed the efficacy of different RET disinfection protocols in reducing the colony-forming unit count or bacterial levels [6, 7].

The present study used MALDI-TOF MS to screen a multitude of viable microorganisms from a cohort of patients with traumatized immature permanent teeth. The rationale of the study was to emphasize the importance of microbial populations identified in cases requiring regenerative endodontic treatment which might influence treatment outcome [1]. The presence of residual infection has been shown to greatly influence the outcome of RET. This has led to the evolution of numerous recent studies attempting to develop novel antimicrobial therapeutics to address this challenge. By being able to precisely define diverse and prevalent microbial populations in traumatized permanent teeth indicated for RET, more effective targeted disinfection and antimicrobial therapies can be designed for regenerative endodontic procedures.

The current study used Bruker Biotyper as it was shown to provide less misidentification when examining the database-absent microorganisms, compared to VITEK MS. The importance of such an advantage is highlighted when this technology is used alone for unusual or difficult microorganism identification [29]. The results of the current study confirmed the diversity and complexity of endodontic infections [30] in such teeth, where 37 isolates were found with reliable identification scores using MALDI TOF MS.

Previous studies conducted on traumatized immature teeth [5, 6] used different methodologies to detect selected or pre-targeted microbiota using selective media culture quantitative analysis or PCR. Viable microorganisms were found in the current study belonging to 25 different species out of 37 isolates, in addition to unexpected types such as *Salmonella typhi, Klebsiella pneumoniae, Bacillus licheniformis, Clostridium sphenoides, and Cupriavidus metallidurans.* The majority of these types were previously identified from root canals in mature teeth, however, mostly used molecular techniques [31–33]. Tzanetakis et al. 2021 [34] studied the prevalence of fungi in primary endodontic infections of a Greek-living population using both Real-time Polymerase Chain Reaction (RTPCR) and MALDI TOF. While they found *Aspergillus* in 8 cases by RT-PCR, no trace of Aspergillus was detected by MALDI-TOF MS. *Candida albicans* was detected in four cases by RT-PCR and identified in two cases by MALDI-TOF MS. They reported significant superiority of PCR in detecting *Aspergillus* only. The inability to discriminate between viable and dead microbiota may explain this superiority. However, the authors performed only aerobic culture while in the present study both aerobic and anaerobic cultures were performed.

Regarding the demographics of the cases in this study, the age range was narrow to minimize variability that may influence results. Traumatized immature permanent teeth were found to be predominant between the age of 10 to 14 years [35]. These teeth are frequently treated by RET to allow for root lengthening and thickening [6].

All cases had an aetiology of trauma with CBCT PAI which scored four and five indicating long-standing challenging infections [36].

Since biofilms are known to penetrate the dentinal tubules of immature permanent teeth as they are greater in number and diameter than in mature teeth [19], the present method of sampling was scraping dentine debris with a sterile file. This method was also used to avoid the difficulties of paper points in reaching the apical third when impregnated with solution [20].

The accuracy of the methodology in the present study was confirmed by performing sterility cultures [5, 6]. Additionally, the streak plate technique used in the present study is used to grow bacteria on a growth media surface so that individual bacterial colonies are isolated and sampled which demonstrates the validity of this technique for microbial culturing [22, 23].

In-office inoculation was performed to ensure recovery of sensitive anaerobes [21], using Thioglycolate broth which allows the differentiation of obligate aerobes, obligate anaerobes, facultative anaerobes, microaerophiles, and aerotolerant organisms [15–17].

Regarding incubation time, it was restricted to 48 h as MALDI TOF MS reliable identification depends upon the stability of bacterial proteins which is decreased by prolonging the incubation time [13, 24]. Furthermore, pure subcultures were performed as MALDI-TOF MS characterization of mono-microbial samples yields highly reliable identification of around 90% of tested samples [13]. This was achieved in the present study in which 92.5% (37/40) of the isolates recovered from the 16 teeth were reliably identified. For accurate assessment of lesion size and to compare with microbial identifications, both volume and CBCT PAI index were calculated as previously shown [37].

Five bacterial phyla were identified in the present study: Firmicutes (59.5%), Bacteroidetes (24.3%), Proteobacteria (10.8%), Actinobacteria (3.7%), and Fusobacteria (3.7%). The prevalence of Firmicutes was statistically significant (*P* < 0.001).These five phyla abundance and Firmicutes prevalence were also reported by Manoharan et al. (2020) [38]who combined culture and molecular sequencing to study thirty traumatic cases in an older age group with a mean of 16.6 years. They additionally found significant differences between the trauma and non-trauma microflora in patients younger than 30 years old.

Monomicrobial infections seen in six cases (37.5%) may have caused the present study’s low mean of 2.5 species per canal despite using state-of-the-art culture technology. This mean was in accordance with Lee et al. 2017 [33] who found the concomitant presence of two (32 teeth) or three species (18 teeth) of bacteria per canal in 50 (80.6%) out of 62 tested teeth with apical periodontitis using selective media plating and biochemical tests in addition to MALDI TOF MS.

The current identified monomicrobial infections may be due to either their antimicrobial activity or their isolation from previously treated canals. Such antimicrobial activity is a recognized feature of all of the three species recovered from monomicrobial primary infections: *Streptococcus constellatus* is known to produce stellalysin which is a relatively large molecular weight (> 10KDa) lytic bacteriocin together with other innumerable small molecular weight (< 6 kDa) bacteriocins commonly produced by the *Streptococcus* genus [39].While *Lactobacillus paracasei* is known to have strong antimicrobial activity against periodontal pathogens such as *S. mutans*, A. *actinomycetem comitans*, *P. gingivalis* and *P. intermedia *[40]. Likewise, *Staphylococcus warneri* is shown to produce bacteriocin nukacin ISK-1 and warnericin RK [41].

*Streptococcus sanguinis* was isolated from a previously treated canal. *S. sanguinis* species produce the bacteriocin sanguicin that inhibits the growth of putative periodontopathic bacteria (PPB) namely: *Porphyromonas gingivalis, Aggregatibacer actinomycetem comitans, Fusobacterium nucleatum, Prevotella intermedia, Tannerella forsythia, and Treponema denticola* [42]. Murad CF et al. in 2014 [43] identified *Streptococcus sanguinis* in 28% of persistent root canal infections, using the checkerboard DNA–DNA hybridization technique.

Regarding the other previously treated canals, the current study was in accordance with previous studies showing monomicrobial prevalence in such canals identifying microbial populations resisting antimicrobial agents [44]. The present study showed two spore bearer bacilli that are resistant to high levels of disinfection: *Clostridium* and *Bacillus* species. These may have been introduced through improperly sterilized dental instruments during previous treatment [45, 46].

The present study periapical lesions were chronic and asymptomatic. This may explain the lack of Red complex (RC) bacteria including *Treponema denticola, Tannerella forsythia* and *Porphyromonas gingivalis*. These bacteria have shown an association with acute and symptomatic periapical disease, while they were rarely identified in chronic and asymptomatic ones [47].

The current study results showed the predominant presence of gram-positive organisms and facultative anaerobes among all microorganisms screened. The reasons for this prevalence may be the absence of coronal sealing in such traumatized teeth which may have altered the path of progression of infection and the shift of the microflora which is expected to occur from initially predominant facultative gram-positive flora to completely anaerobic gram-negative bacteria which occurs after closure of these teeth [48]. Furthermore, contact with the oral environment and gingival crevices via pulp exposure, cracks or only exposed dentinal tubules has been considered the main route of invading microbes in traumatized teeth [19]. The results of the current study were in accordance with Marcotte and Lavoie, 1998 [49] who showed the predominance of facultative anaerobic bacteria, particularly gram-positive in healthy gingival crevices. The present study results coincide with Ferreira et al. (2006)[50 who identified the incidence of strict anaerobes by 19% and facultative anaerobes by 81%, with the predominance of gram-positive species (75.8%) in the canals of dogs opened for 120 days showing periapical lesions. Furthermore, the most frequently isolated Genus was *Streptococcus*similar to the current study. *Streptococcus* was previously reported as a strong producer of extracellular proteins capable of inducing apical periodontitis [51].The predominance of Gram-positive facultative anaerobes was also found with coronal leakage of root-filled teeth showing persistent periapical lesions by Adib et al. 2004 [52] again coinciding with the present study.

*Enterococcus faecalis* was the current most prevalent species which has been similarly found in mature teeth [53], yet this was differently shown by Baumotte et al. 2011 [5] where he found it in 5.25% of the immature teeth studied.

Interestingly, the current study results were able to discriminate between *S. pneumoniae* and *S. mitis* in the same case despite the great similarity between alpha-hemolytic (viridans) *streptococci*, the similar nucleotide sequences of the 16S rRNA genes from *S. mitis & S. pneumonia* and their similar mass spectra. Marin et al. 2017 [54] detected several minor mass peaks allowing for such discrimination. Hence, MALDI-TOF was suggested as a tool for the rapid identification of *S. pneumoniae* [55]. On the contrary to the present identification of *S. pneumoniae,* Nandakumaret al in 2008 [56] attempted to identify *S. pneumoniae* to prove anachoresis of respiratory bacteria, however, they did not identify it from root canals with PCR-based methods. Yet, the current study identification of such organisms could not again support anachoresis. This may be due to the fact that existing traumatized fractured crowns may have been predisposed to the presence of micro or macrocracks as more obvious routes of microbial contamination.

Furthermore, the present identification of *Cupriavidus metallidurans* from the root canal of a previously hospitalized patient after maxillofacial surgery coincides with Langevin et al. in 2011 [57] who reported *C. metallidurans*; repeatedly cultured from the blood of a patient with multiple complications after invasive surgery. Moreover, two strains of *C. metallidurans* were also identified from the respiratory secretions of cystic fibrosis patients [58].

Regarding the comparison between microbial identifications in accordance with the size of the lesions in the CBCT, no significant differences were found. While it has been shown that long-standing infections, and larger-sized lesions harbor more and diverse microorganisms [36] this was not found in the current study. However, it may have been highlighted by the statistically significant difference between CBCT PAI scores according to gram stain. Mixed gram-negative and positive infections scored five while gram-positive infections alone scored both four and five. This was in accordance with Martinho et al. in 2010 [59] who found a correlation between the number of gram-negative bacteria and the levels of Interleukin IL-1beta/ Tumor Necrosis Factor TNF-alpha which were related to the larger size of the radiolucent area and to the antigenicity of the endodontic contents.

While the present study included a similar number of cases to those of Baumotte et al.,2011 [5] and Nagata et al., 2014 [6], however, a limitation of the current study is that cases included different clinical manifestations and periapical diagnoses which made a comparison of the results based on these criteria difficult. The present study offers some additional insight into the diversity and prevalence of microorganisms identified in immature necrotic teeth, however, other limitations were present including the potential existence of unculturable organisms and the potential existence of culturable organisms in a viable but nonculturable (VBNC) state. An additional possible limitation is that of the MALDI TOF MS microbial software Bruker DB 4613 to have a complete genus/species list. The lack of sufficient spectra in the database may lead to lack of identification or misidentification.

## Conclusion

The microbiota of traumatized immature necrotic permanent teeth is complex, diverse, and predominantly polymicrobial with a significant predominance of Gram-positive facultative anaerobes. MALDI-TOF MS can provide rapid and accurate identification of viable endodontic microbiota thereby it can potentially guide appropriate disinfection procedures before RET. The adoption of irrigation and intra-canal medicaments effective against such microbial types during RET presents the current study's translational value/clinical implication and the future perspective.

## Data Availability

The datasets during and/or analysed during the current study are available from the corresponding author on reasonable request.

## Abbreviations

MALDI-TOF MS: Matrix-Assisted Laser Desorption/Ionization Time-Of-Flight Mass Spectrometry
CBCT PAI: Cone beam computed tomography periapical index
RET: Regenerative endodontic treatment
SCAPs: Stem cells from the apical papilla
TAP: Triple antibiotic paste
CHP: Calcium hydroxide with chlorhexidine
mNGS: Metagenomic next generation sequencing
PMF: Peptide mass fingerprinting
MS: Mass spectra
STROBE: STrengthening the Reporting of OBservational studies in Epidemiology
RTPCR: Real-time Polymerase Chain Reaction
PPB: Putative periodontopathic bacteria
RC: Red complex
IL-1 beta: Interleukin
TNF-alpha: Tumor Necrosis Factor
VBNC: Viable but nonculturable
